# Multidisciplinary lifestyle treatment for type 2 diabetes in 12 European countries: protocol for a quasi-experimental study

**DOI:** 10.1186/s12889-025-22246-w

**Published:** 2025-03-19

**Authors:** Isabel Diez-Valcarce, Marta M. Pisano-González, Cristina Fernández García, Jaana Linstrom, Jelka Zaletel, Claudia Giacomozzi, Foetini Tolika, Inés Rey Hidalgo, Alberto Lana

**Affiliations:** 1 Regional Health Service of the Principality of Asturias (SESPA), Plaza del Carbayon 1,2, Oviedo, Asturias 33017 España; 2 Ministry of Health of the Principality of Asturias (CSPA), Calle Ciriaco Miguel Vigil, 9, Oviedo, 33005 España; 3 Health Research Institute of the Principality of Asturias (ISPA), Avda. del Hospital Universitario, Oviedo, s/n, 33011 España; 4 Department of Public Health, National Institute for Health and Welfare, POB 30, Helsinki, 00271 Finland; 5 Institute of Public Health and Clinical Nutrition, University of Eastern Finland, POB 1627, Kuopio, 70211 Finland; 6 National Institute of Public Health, Trubarjeva cesta 2, Ljubljana, 1000 Slovenia; 7 Department of Cardiovascular and Endocrine-Metabolic Diseases and Aging, The Italian National Institute of Health, Viale Regina Elena 299, 00161 Rome, Italy; 8 Directorate of Public Health, 1st Regional Healthcare Authority of Attica, Ministry of Health, 18, Valaoritou Str., Athens, 10671 Greece; 9 Foundation for the Promotion of Applied Scientific Research and Technology in Asturias (FICYT), Calle Cabo Noval, 11-1C, Oviedo, 33007 España; 10 Department of Medicine, Faculty of Medicine and Health Sciences, University of Oviedo, Avda. Julián Clavería s/n, Oviedo, 33006 España

**Keywords:** Diabetes mellitus, Type 2. health education, Healthy lifestyle, Selfcare, Secondary prevention

## Abstract

**Background:**

The incidence and prevalence of type 2 diabetes (T2DM) are expected to continue rising. T2DM causes life-threatening, disabling and costly complications, and significantly reduces quality of life and life expectancy. The burden of T2DM can be reduced using comprehensive lifestyle modifications. The aim of this study is to evaluate the applicability and cost-effectiveness of a multicomponent, multidisciplinary lifestyle program in 22 European regions and to generate guidelines for transfer to European health care systems.

**Methods:**

A quasi-experimental study (without a control group) will be conducted to evaluate the CARE4DIABETES program, which is based on the Reverse Diabetes 2Now best practice. The program will involve more than 120 healthcare professionals and 860 people with T2DM from 12 European countries - Belgium, Bulgaria, Finland, Hungary, Italy, Greece, Malta, Poland, Portugal, Slovakia, Slovenia and Spain. Patients will be enrolled based on clinical criteria and motivation for change. The program will have two phases, an intensive phase (6 months) with face-to-face and online training to achieve behavioral change, and an online aftercare phase (6 months) to consolidate changes. The program will be evaluated for impact, sustainability and cost-effectiveness using a combination of validated questionnaires at baseline, six months and one year after the start of the intervention.

**Clinical trial number:**

Trial registration number: ISRCTN62063346.

## Introduction

Type 2 diabetes mellitus (T2DM) is a highly heterogeneous non-communicable chronic disease with a variable age of onset, associated with an undetermined degree of obesity and insulin resistance, and with tendency to develop health- and life-threatening complications [1]. Widespread worldwide, T2DM is already one of the major public health challenges for middle- and high-income countries [2]. Although the underlying causes of T2DM are a puzzle with genetic, physiological, and behavioural pieces -some of which remain unknown-, probably lifestyle-related factors are the dominant cause. Therefore, a comprehensive approach for people living with T2DM should pursue long-lasting lifestyle modifications [3].

There is robust evidence on the efficacy of a wide range of interventions to improve glycaemic control and self-management disease via changes in relevant health-related behaviours, including adherence to pharmacological treatment and to healthy lifestyle, which theoretically lead to long term improvements in T2DM progression [4]. Some examples of effective interventions include among the others: programs to enhance health literacy, disease information and skills of e-health strategies, such as gamification, use of wearable devices or telemonitoring; peer-led interventions; mind practices to reduce stress [5–14].

There is also a growing body of evidence for T2DM remission, that can be achieved through intensive behavioural interventions, alone or combined with bariatric surgery or pharmacological interventions [15–17]. T2DM remission is considered when maintaining glycated haemoglobin below 6.5% without pharmacological treatment for three months [18], and it has been recognized by the World Health Organization and scientific associations as an appropriate therapeutic goal [19]. However, according to the American College of Lifestyle Medicine, therapeutically dosed intensive lifestyle modifications should be preferentially recommended as the primary modality for T2DM care and remission [20]. Interventions should include personalized nutrition coupled with physical activity engagement as ways to reach weight loss [19]. However, long-term weight regain is the usual hurdle encountered by patients and clinicians in the real-world setting, as the difficulty of long-lasting restricted diets, such as very low-calorie or nutritional ketosis diets, hinders the sustainability of any potential results. Therefore, studies targeting different populations on the efficacy of various complementary strategies for weight management, including mental stress and sleep disorders [21], are required [19]. Additionally, the American Diabetes Association has introduced another recommendation for the inclusion of technology assisted T2DM programmes based on patients’ preferences [22].

Against this background, a multicomponent and multidisciplinary programme called “Reverse Diabetes 2Now” (RD2N) was developed in The Netherlands seeking remedy for T2DM [23]. RD2N focuses on improving participant skills -rather than just knowledge- to manage relevant components of their lifestyle, regaining control over their disease. In essence, RD2N is a 6-month programme that provides intensive group-based counselling on nutrition, physical activity, sleep and stress management, biometric feedback, and cooking classes, combined with a digital coaching platform and close physician-monitoring of medication use. In preliminary studies, RD2N has demonstrated lasting real-life benefits, especially in terms of body weight, medication use and quality of life in people living with T2DM [24].

RD2N was selected by the European Union as best practice in primary care, to be tested as a joint action in different countries. CARE4DIABETES - Reducing the burden of non-communicable diseases by providing a multi-disciplinary lifestyle treatment intervention for T2DM - was the name given to the joint action. Therefore, CARE4DIABETES (C4D) was conceived as a comprehensive and structured framework to design, adapt, implement and evaluate the RD2N best practice in the EU, seeking for a sustainable reduction in the burden of T2DM and its related risk factors, both at societal and personal level, as well as raise awareness and acceptance on improved and more innovative related lifestyle interventions, in line with EU policy framework and the Action Plan for Prevention and Control of Non Communicable Diseases in the World Health Organization (WHO) EU Region 2016–2025 [25].

This paper describes the study protocol of C4D to transfer the RD2N best practice into 12 selected European countries: Belgium, Bulgaria, Finland, Hungary, Italy, Greece, Malta, Poland, Portugal, Slovakia, Slovenia, and Spain. Conclusions about acceptance and effectiveness in different contexts will allow the production of guidelines for transferability of the intervention in the EU.

## Methods

### Design and ethical considerations

C4D will be a one-group pre-test/post-test quasi-experimental study, conducted in concert in 12 countries. Briefly, C4D will evaluate the acceptability and efficacy of an innovative multicomponent intervention addressing several lifestyle behaviours (i.e. nutrition, physical activity, sleep, and stress management), that can trigger the achievement of long lasting body weight control, healthy glucose levels, lowering -and in some cases interrupting- medication consumption, reducing comorbidities, complications and associated healthcare costs, and improving health-related quality of life. The intervention will be based on the “Integrated Model for explaining motivational and behavioural change” (I-CHANGE model) and will focus on improving participants’ skills - not just knowledge - to manage relevant components of their lifestyle [26]. The structured implementation process, including training and capacity building of healthcare personnel and cultural adaptation according to local situation, aims to produce a sustainable lifestyle care model that can be scaled up nationally. Each country/partner will have a local multidisciplinary team in charge of driving the process, consisting of at least a diet expert, registered nurse or general practitioner, a coach, facilitator or equivalent and a lead coordinator (Table 1). The C4D will run from February 2023 to January 2026.

**Table 1:** Roles and functions of the intervention team by voeding Leeft foundation

Role	Responsibilities in RD2N practice	
Diet expert	• Physiological explanation of the metabolic disturbance of type 2 diabetes (insulin resistance). • Guidance for participants in changing eating habits.	
Registered Nurse or General Practitioner	• Safe process guidance for individual participants. • Individual and group guidance on reduction of medication and reversing type 2 diabetes. • Manage participant record and registration. • Contact with the lead practitioner of the participant.	
Coach/facilitator or equivalent	• Create a safe setting for the individual and the group. • Activate and monitor group dynamics. • Guidance in awareness and behavioural change. • Guide to activation and integration of sustainable behaviour.	
Coordinator	• Quality assurance. • Practical organization of the program • Communication with participants. • Administration participants and keeping overview of progress.	

The study has been approved by an ethics committee (EC) in each participating country to conduct the activities in accordance with EU ethical standards. Study participants must sign the informed consent form before starting the proposed activities. Previously, they will have received, through an information sheet and interview with the assigned professional of reference, all the related information, as to resolve any doubt that may arise beforehand. Since behavioural changes are expected from the beginning of the program, the first pharmacological reduction of antidiabetic agents should be carried out early during the first intervention sessions unless EC and/or clinicians recommended differently for a specific country. Further medication modifications will be made during the program based on the biometric levels of the participants. Thereby, C4D will be implemented under strict (and continuous) medical supervision.

### Stages/Sequencing actions

**Fig. 1:**
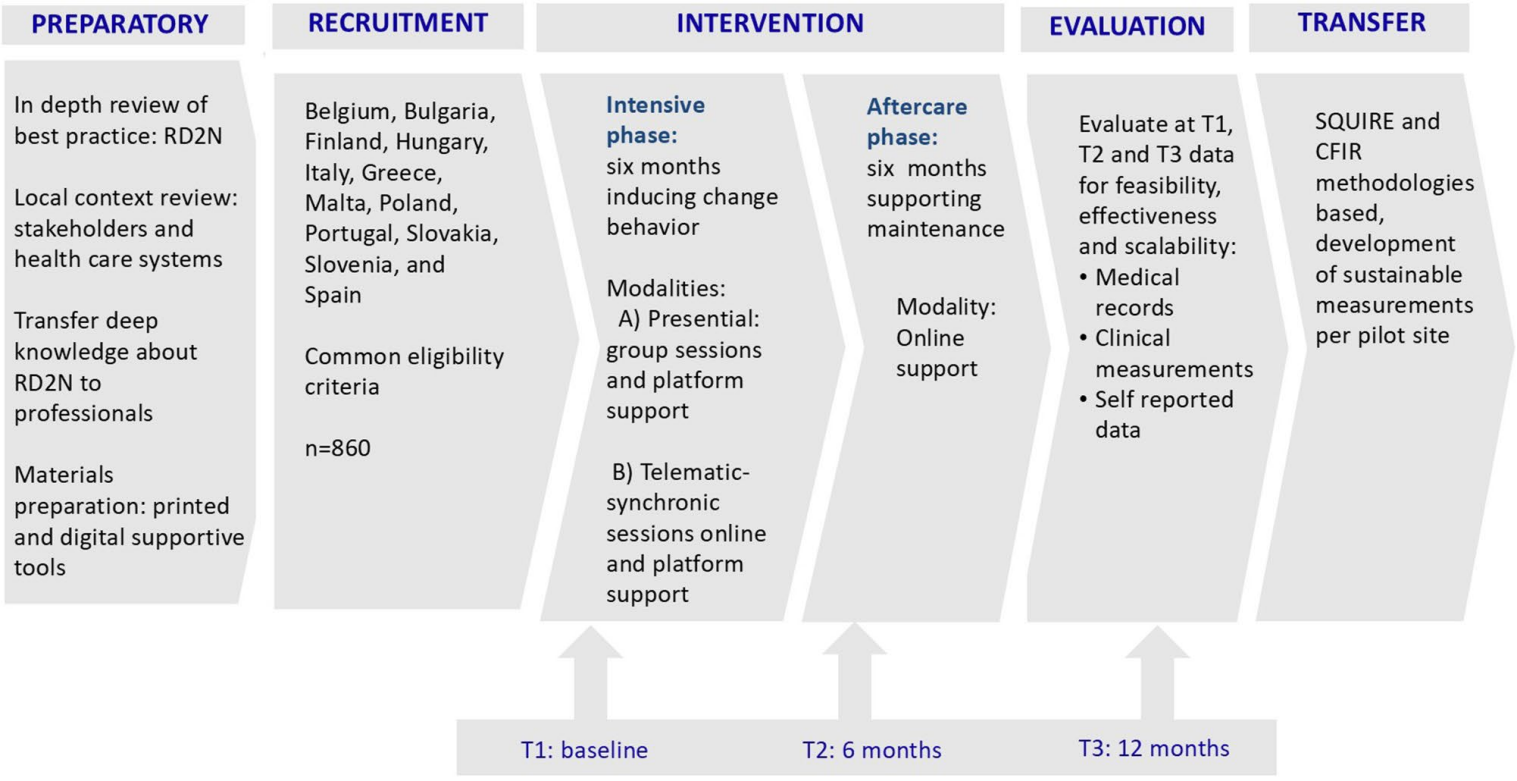
Sequence of stages of C4D

The study protocol is divided in different stages and each of them applies different approaches to ensure its scientific soundness (Fig. 1).

#### Preparatory actions

The challenge of transferring a particular contextualized successful practice into a different environment along the EU requires a deep knowledge of the original RD2N best practice. But at the same time, the uniqueness of each context needs to be analysed to provide the best opportunity to transform RD2N best practice into a local real possibility. Therefore, preparatory actions include a comprehensive analysis of general aspects to be considered by the local teams when adapting, implementing, monitoring, and evaluating the RD2N best practice. They also involve the examination of country-specific circumstances, including key stakeholders’ identification as local facilitators of the entire process. Different methodologies will be applied: (a) SCIROCCO [27], self-assessment tool which will be used to identify the maturity of the health and social care systems, for the adoption and scaling up of integrated care, or best practice solutions; (b) Consolidated Framework for Implementation Research (CFIR), a construct to analyse determinants [28]; it is designed to describe barriers and facilitators to implementation outcomes; (c) SQUIRE 2.0, a framework for reporting new knowledge about the actions and how to improve healthcare [29]; (d) SWOT analysis (acronym of strengths, weaknesses, opportunities and threats), a technique for assessing the national, regional and local contexts, including stakeholders [30], which can help to analyse what each partner does best now, and to devise a successful strategy for the future.

During this preliminary stage, up to 120 healthcare professionals (i.e. up to 10 *per* country) are directly trained by Dutch RD2N best practice owner for their actions in their countries. The training is designed online over 5 sessions with all selected members of the multidisciplinary team per each country (together), that will receive the “know how” to transmit to people with T2DM the fundamental elements of the intervention. Three formations have been planned with four countries in each formation to effectively deliver the training. Then, local multidisciplinary team members will adapt the content and materials to their respective local languages and context. The main materials that need adaptation are: the participant’s book, recipe book, formative content of the intervention sessions and entries on the digital platform.

#### Recruitment of participants

People with a physician-diagnosed T2DM will be invited by the healthcare worker in charge of their follow-up, who will previously determine the compliance with selection criteria. A candidate will be considered for participation if he/she meets all eligibility criteria (Table 2). People already selected must be informed in detail about their participation and commitments and signed the consent form; they must fill in baseline registration forms to be compared with new forms required as their participation progresses.

**Table 2:** Eligibility criteria for participants

Age 20–80 years
T2DM treated with medication (oral or injected medicines or insulin)
T2DM duration 1–10 years
BMI 25–40 kg/m^2^
No COPD or kidney or heart failure diagnosis
No bariatric surgery (self-reported/medical record)
No eating disorder (self-reported /medical record)
No pregnancy (self-reported /medical record)
Committed to make lifestyle changes to control T2DM
Ability to use necessary digital devices
Access to internet
Sufficient language skills to take part in the program
Possibility to take part in the program as provided (schedule, location)
Willingness to measure blood glucose at home

T2DM: type 2 diabetes mellitus, BMI: body mass index; COPD: chronic obstructive pulmonary disease

**Fig. 2:**
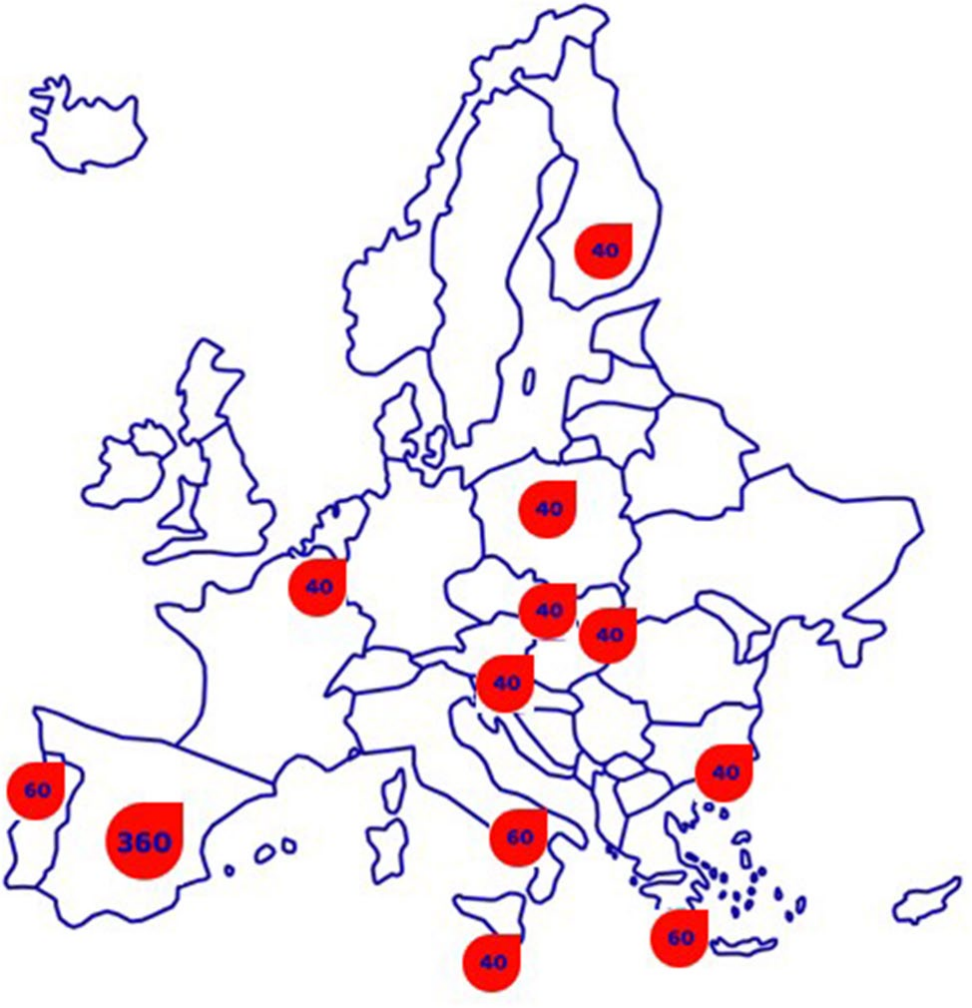
Minimum sample sizes per countries

According to C4D study protocol, 860 people living with T2DM are expected to be recruited, with a minimum of 40 participants *per* country (Fig. 2). An estimation was done considering body weight as main outcome. Based on the 6-month body weight reduction results from the original best practice, with standard deviation of 5.1 kg, we can estimate that a sample size of 40 participants is sufficient for a paired sample t-test (power of 80%, type 1 error rate 0.05), assuming the true effect size (mean reduction in body weight during the first 6 months) is 2.3 kg. Therefore, 860 participants will be enough to test the hypothesis involving health-related quality of life.

#### Intervention

Although C4D adapts the intervention features to country-specific guidelines, contexts and needs, there is an overall approach to achieve a robust, structured framework. The intervention consists of a T2DM self-management education and support group program [31], based on the I-Change model [26], and developed over 12 months, the program consists of two phases: an intensive phase followed by an aftercare intervention, each lasting 6 months.

**Fig. 3:**
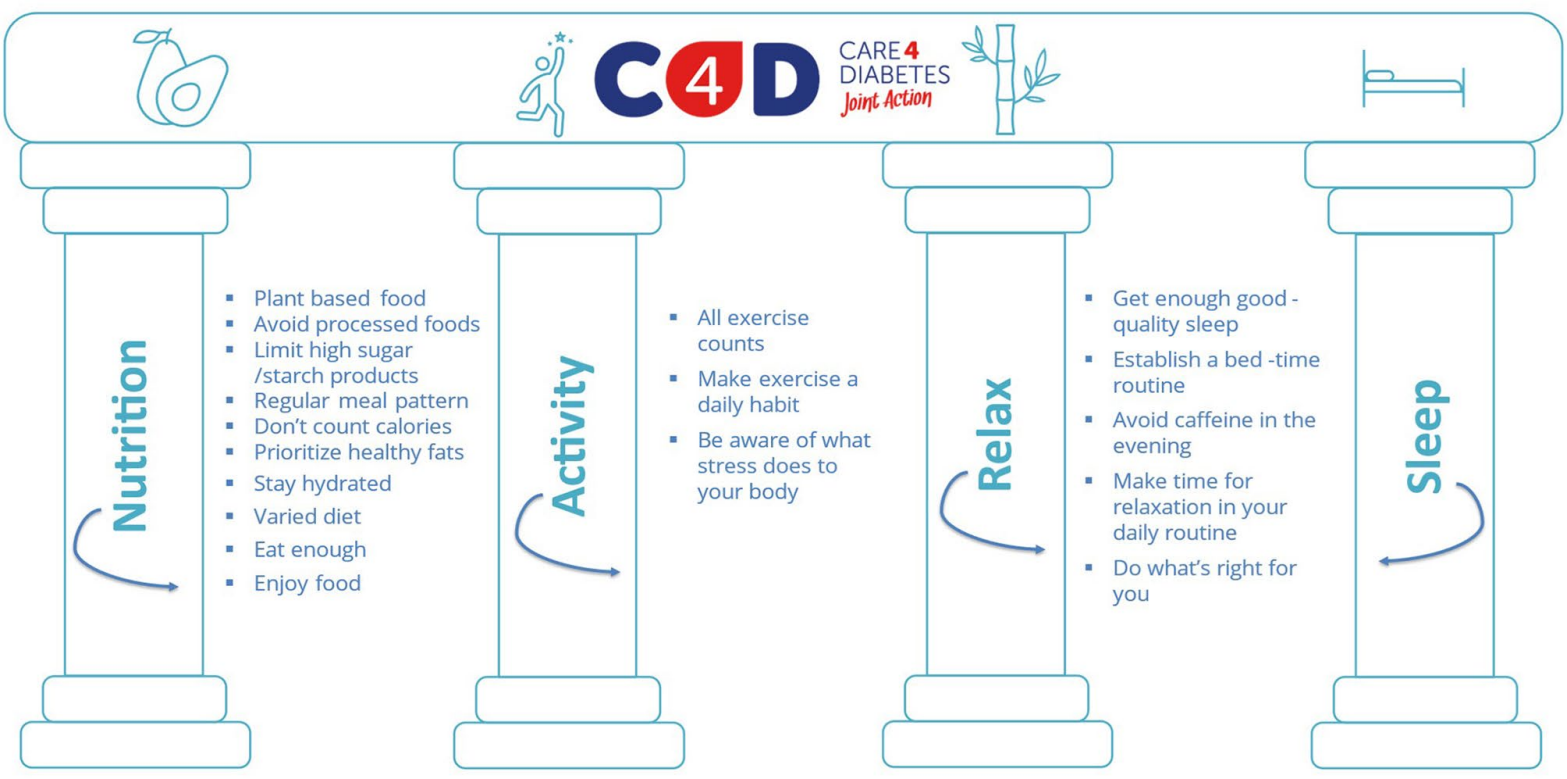
Pillars of C4D intervention

The intensive phase provides participants with theoretical content and practical activities on the 4 pillars - nutrition, physical activity, sleep and relaxation - of RD2N best practice (Fig. 3). Activities and workshops related to this content include interpretation of changes in glucose levels throughout the day, biometric feedback, physical exercises, relaxation and coaching practices, and cooking classes. C4D provide people living with T2DM with skills for incorporating healthy habits into their lifestyles in an experiential dynamic way (i.e. participants must ‘live’ and practice the concepts they receive during the theoretical sessions). For pedagogic and organizational reasons people living with T2DM will be organized into groups of 20 people approximately.

The intensive phase tests two different delivery modes with identical content, one face-to-face and the other telematic, supported by a digital platform in both cases. The face-to-face modality refers to interventions consisting of about 6 on-site group training sessions over 6 months. A novel aspect of C4D is the inclusion of an overnight stay in order to: (a) promote group bonding, through joint acquisition of the knowledge received and its practice; (b) focus attention, by taking participants out of their own environment, responsibilities and situation at home; (c) aid follow-up, as the stay would allow for greater control over program follow-up (fewer triggers in the program environment, such as e.g., snacking); (d) guarantee for safety, as participants would feel physically and emotionally safe (especially participants on hypoglycaemic medication); (e) ease the burden, as the two start days are fully scheduled and intensive. Conversely, the telematic modality refers to several fully digital group sessions. The online platform will offer support throughout the duration of both intervention modalities, including coaching, close personal advice, and physician-monitoring of medication use.

The aftercare intervention over the following 6 months ensures that barriers are removed, and new habits become sustainable over time. Integrating T2DM self-care into daily life is a challenge for participants and their families. Therefore, individual participant characteristics, social determinants of health, and psychosocial factors should be part of the person-centered care, respecting, and responding to their individual preferences and barriers [1].

#### Evaluation

C4D includes three types of evaluation: impact, process and economic evaluation.

The impact of the intervention will be assessed in terms of behavioural change, participant clinical characteristics related to people living with T2DM (anthropometric measures, biomarkers, medication use, etc.) and quality of life. Measurements will be taken at three time points: T1: baseline (prior to the beginning of the intervention); T2: between the intensive and aftercare phases (6 months); and T3: immediately after the end of the intervention (12 months). Trained staff using standardised methods will collect study variables, like laboratory and clinical measurements, and self-report questionnaires (Table 3); agreed validated questionnaires will be requested and made available when appropriate. The survey will be available in English and needs to be translated into local languages. The translation and translation-back process and contextual adaptation will be used to ensure compatibility with the original questionnaires adapting to regional characteristics.

**Table 3:** Clinical and behavioural measurements

Measurements	Timeline			Data collection method
	T1: 0 baseline	T2: 6 months	T3: 12 months	
Sociodemographic characteristics)	x			Self-reported questionnaire
Lifestyle behaviors (smoking, diet pattern, alcohol intake)	x			Self-reported questionnaire
T2DM duration	x			Medical record
Height	x			Clinical measurement
Weight	x	x	x	Clinical measurement
Waist circumference	x	x	x	Clinical measurement
Blood pressure	x	x	x	Clinical measurement
Serum lipids	x	x	x	Laboratory test
Glycosylated hemoglobin	x	x	x	Laboratory test
Medication for diabetes	x	x	x	Medical record
Medication for blood pressure and lipids	x	x	x	Medical record
Quality of life	x	x	x	Self-reported questionnaire
Perceived health	x	x	x	Self-reported questionnaire
Self-care for chronic diseases	x	x	x	Self-reported questionnaire
Self-efficacy	x	x	x	Self-reported questionnaire
Fatigue	x	x	x	Self-reported questionnaire
Sleep problems	x	x	x	Self-reported questionnaire
Physical activity and muscle-strengthening	x	x	x	Self-reported questionnaire
Diet (food frequency questionnaire)	x	x	x	Self-reported questionnaire

The process evaluation considers the satisfaction and experience of participants and staff, using a self-reported questionnaire. The evaluating process follow the RE-AIM framework (Table 4). In addition to the quantitative parameters, qualitative data collect factors influencing participation/non-participation; elements contributing the outcomes; the barriers preventing adoption (both by care providers and participants); how was the intervention implemented; and what will be sustained, discontinued, or adapted, and why.

**Table 4:** Components of RE-AIM framework

Component	Indicator
Reach	
*Inclusion*	Activities/procedures to attract possible participants
	Number of potential participants screened for eligibility
*Exclusion*	Number of potential participants excluded (per exclusion criterion)
*Background characteristics*	Characteristics of participants compared to non-participants or to target population
Effectiveness	
	Number of sessions/hours of intervention organized
	Number of intervention sessions/hours of intervention attended by each participant
	Attrition (%)
	Outcome measures (%) completed at each timepoint
Adoption	
	Characteristics of settings participating compared to non- participants
	Number (and %) of staff participating
	Characteristics of staff participants vs. typical staff
Implementation	
	Adaptations made to intervention (Phase 1)
	Cost of intervention
	Consistency of implementation across staff/settings
Maintenance	
	Outcome measures at 12 months
	Adaptations made to intervention (Phase 2)
	Model integration to the organization

Socio-demographic characteristics such as sex, age, education, immigrant background, cohabitation and income level will be analysed.

C4D will collect data on the costs, and the economic impact of the implemented practice, comparing the intervention costs against the benefits of the practice (e.g. quality of life measured with the validated questionnaire). Economic data collection will include the transfer and adaptation costs of the program per country and the implementation phase. Transfer and adaptation data will include developing the online platform, translating and adapting the materials, planning the practicalities of the intervention and training the trainers. The implementation costs will include labour (cost of training staff to deliver the intervention and the personnel cost of the actual intervention), capital (the cost of renting spaces to undertake practice’ activities), administrative (the cost of promoting the intervention to eligible participants), consumables (other materials, tools, goods needed for the intervention implementation) and overhead costs.

#### Quality control

Two different tools, proven effective during previous European projects are included to assure quality control. During the intervention, PDSA methodology [32], acronym of “plan, do, study and act” ensures to make corrections and fine-tune the design for future use. The second tool will be the Standards for Quality Improvement Reporting Excellence (SQUIRE) [33], which are a set of 19 descriptive units that present how to examine and develop quality improvements in healthcare. The guidelines were developed over the years to respond to the insufficient and limited studies published in this field. They have proven to be useful designing implementation, and for writing communications.

#### Transfer

The Consolidated Framework for Implementation Research (CFIR) [28] will be used to examine factors influencing different levels of care delivery (patient care, delivery groups, health organisation or policy) that could hinder or facilitated the implementation of the best practice in C4D. The CFIR offers some constructs that have proven effective during the implementation. It is adaptable to a variety of contexts and settings, and covers five key domains: the intervention, the internal and external environment, the people involved, and the implementation process itself. Each domain comprises multiple constructs that interact in complex ways to influence the effectiveness of implementation. CFIR provides the multidisciplinary teams with a specific methodology to identify relevant factors influencing implementation and increasing their potential success rate for future implementations.

## Discussion

C4D will be highly relevant to the EU 4 H Programme [34], which sets as one of their key priorities the decrease in the impact of non-communicable diseases on individuals and on society. Based on RD2N effective lifestyle treatment for people with T2DM and the underlying principles of behavioural change (I-Change model) [26], the C4D purpose will be to improve and foster health in the EU Member States by reducing the burden of T2DM and its related risk factors and complications, both at societal and personal level.

Adopting general healthier habits in life can have wide-reaching positive effects on various health parameters as they are interconnected, and positive changes in one area often contribute to improvements in others like cardiovascular, respiratory, and other non-communicable diseases: bone health, stronger immune and cognitive function. Although the concept of T2DM remission has emerged as a real-world option, effective implementation in routine clinical practice may not be feasible until long-term studies prove the efficacy of different approaches [35] and the different European context testing the Dutch RD2N practice will contribute to those efforts. The common peer groups will be a novel contribution to enhance diabetes knowledge, reinforce each other, and receive support for change in behaviour [4], adding mental and social well-being to the results.

Improved lifestyle, and risk factor management among people with T2DM may reduce costs related to T2DM treatment, and in the long term, costs related to reduced functional and working capacity [36] and to the management of T2DM complications. The diversity on the socioeconomic context, healthcare systems, and the level of commitment from the 12 countries participating demand tailored approaches to consider the unique characteristics and challenges of each one of them. It underscores the relevance of collecting data and insights from various sources to inform successful implementation strategies in different scenarios.

The absence of a control group in the study limits the ability to draw strong conclusions or causal claims about the effects of the intervention. These actions will test the feasibility and practical aspects of the intervention, and control groups should be introduced in subsequent studies once the C4D is over, for the purpose of attributeing observed changes or outcomes truly because the intervention and not to other factors that might influx.

The results of C4D are expected to support new EU policies, as all countries see their affected population increase and seek for a sustainable reduction in the overall burden of T2DM and its related risk factors and complications.

## Data Availability

No datasets were generated or analysed during the current study.

## Declarations

**Table:**

APPROVAL DATE*	ETHICAL COMITTEE NAME
Belgium 20/09/2023	University Ghent (Ugent) and Ghent University Hospital (UZ Gent)
Bulgaria 30/11/2023	Research Ethics Committee Southwestern University “Neofit Rilski”
Finlandn 5/11/2023	HUS Regional Committee on Medical Research Ethics
Greece 21/09/2023	Ethics Comittee of General Alexandra Hospital
Hungary 07/01/2025	Medical Research Council (Egészségügyi Tudományos Tanács)
Italy 24/10/2023	CEN (Comitato Etico Nazionale)
Malta 05/03/2024	Health Ethics Committee
Poland 13.11.2023	Bioethics Committee at Medical University of Warsaw
Portugal 17/2/2024	APDP local EC
Portugal 6/02/2024	Gabinete de Projetos de Investigação Centro Académico Clínico ICBAS- CHUdS
Portugal 29/04/2024	Comissão de Ética para a Saúde da ARSLVT
Portugal 16/01/2024	Comissão de Ética da ULS Baixo Alentejo
Portugal 15/01/2024	Comissão de Ética da ARS Algarve
Slovenia	Komisija za deonotološka in etična vprašanja - KDEV (Commission for Deontological and Ethical Issues)
Slovakia 03/11/2023	Etická komisia Národného endokrinologického a diabetologického ústavu n.o.
Spain 11/10/2023	Comité de Ética de la investigación con medicamentos del Principado de Asturias

## References

[1] Davies MJ, Aroda VR, Collins BS, Gabbay RA, Green J, Maruthur NM, et al. Management of hyperglycemia in type 2 diabetes, 2022. A consensus report by the American diabetes association (ADA) and the European association for the study of diabetes (EASD). Diabetes Care. 2022;45(11):2753–86. 36148880 10.2337/dci22-0034 PMC10008140

[2] Khan MAB, Hashim MJ, King JK, Govender RD, Mustafa H, Al Kaabi J. Epidemiology of type 2 Diabetes– Global burden of disease and forecasted trends. J Epidemiol Glob Health. 2019;10(1):107. 10.2991/jegh.k.191028.001 PMC7310804 32175717

[3] Caturano A, Galiero R, Rocco M, Tagliaferri G, Piacevole A, Nilo D, et al. Modern challenges in type 2 diabetes: balancing new medications with multifactorial care. Biomedicines. 2024;12(9):2039. 39335551 10.3390/biomedicines12092039 PMC11429233

[4] Asmat K, Dhamani K, Gul R, Froelicher ES. The effectiveness of patient-centered care vs. usual care in type 2 diabetes self-management: A systematic review and meta-analysis. Front Public Health. 2022;28:10:994766. 10.3389/fpubh.2022.994766 PMC9650641 36388341

[5] Butayeva J, Ratan ZA, Downie S, Hosseinzadeh H. The impact of health literacy interventions on glycemic control and self-management outcomes among type 2 diabetes mellitus: A systematic review. J Diabetes. 2023;15(9):724–35. 37407516 10.1111/1753-0407.13436 PMC10509520

[6] Ossenbrink L, Haase T, Timpel P, Schoffer O, Scheibe M, Schmitt J, et al. Effectiveness of digital health interventions containing game components for the Self-management of type 2 diabetes: systematic review. JMIR Serious Games. 2023;11:e44132. 37261900 10.2196/44132 PMC10273035

[7] Cai J, Xu H, Jiang S, Sung J, Sawhney R, Broadley S, et al. Effectiveness of telemonitoring intervention on glycaemic control in patients with type 2 diabetes mellitus: A systematic review and meta-analysis. Diabetes Res Clin Pract. 2023;201:110727. 37217016 10.1016/j.diabres.2023.110727

[8] Verma I, Gopaldasani V, Jain V, Chauhan S, Chawla R, Verma PK, et al. The impact of peer coach-led type 2 diabetes mellitus interventions on glycaemic control and self-management outcomes: A systematic review and meta-analysis. Prim Care Diabetes. 2022;16(6):719–35. 36307372 10.1016/j.pcd.2022.10.007

[9] Sanogo F, Xu K, Cortessis VK, Weigensberg MJ, Watanabe RM. Mind- and Body-Based interventions improve glycemic control in patients with type 2 diabetes: A systematic review and Meta-Analysis. J Integr Complement Med. 2023;29(2):69–79. 36070591 10.1089/jicm.2022.0586 PMC13134557

[10] Luo J, Zhang K, Xu Y, Tao Y, Zhang Q. Effectiveness of wearable Device-based intervention on glycemic control in patients with type 2 diabetes: A system review and Meta-Analysis. J Med Syst. 2022;46(1):11. 10.1007/s10916-021-01797-6 34951684

[11] Othman MM, Khudadad H, Dughmosh R, Syed A, Clark J, Furuya-Kanamori L, et al. Towards a better Understanding of self-management interventions in type 2 diabetes: A meta-regression analysis. Prim Care Diabetes. 2021;15(6):985–94. 34217643 10.1016/j.pcd.2021.06.006

[12] Avery L, Flynn D, Van Wersch A, Sniehotta FF, Trenell MI. Changing physical activity behavior in type 2 diabetes. Diabetes Care. 2012;35(12):2681–9. 23173137 10.2337/dc11-2452 PMC3507564

[13] Seo HJ, Kim SY, Sheen SS, Cha Y. e-Health interventions for Community-Dwelling type 2 diabetes: A scoping review. Telemed E-Health. 2021;27(3):276–85. 10.1089/tmj.2019.0263 32552559

[14] Kim J, Hur MH. The effects of dietary education interventions on individuals with type 2 diabetes: A systematic review and Meta-Analysis. Int J Environ Res Public Health. 2021;18(16):8439. 34444187 10.3390/ijerph18168439 PMC8393495

[15] Li M, Jeeyavudeen MS, Arunagirinathan G, Pappachan J. Is type 2 diabetes mellitus a behavioural disorder?? An evidence review for type 2 diabetes mellitus prevention and remission through lifestyle modification. Eur Endocrinol. 2023;19(1):7. 10.17925/EE.2023.19.1.7 PMC10258624 37313234

[16] Balasubaramaniam V, Pouwels S. Remission of type 2 diabetes mellitus (T2DM) after sleeve gastrectomy (SG), One-Anastomosis gastric bypass (OAGB), and Roux-en-Y gastric bypass (RYGB): A systematic review. Med (Mex). 2023;59(5):985. 10.3390/medicina59050985 PMC10222088 37241216

[17] Hao S, Umpierrez GE, Vellanki P. Intervention with therapeutic agents, Understanding the path to remission to type 2 diabetes. Endocrinol Metab Clin North Am. 2023;52(1):39–47. 36754496 10.1016/j.ecl.2022.07.004 PMC10158502

[18] Riddle MC, Cefalu WT, Evans PH, Gerstein HC, Nauck MA, Oh WK, et al. Consensus report: definition and interpretation of remission in type 2 diabetes. Diabetes Care. 2021;44(10):2438–44. 34462270 10.2337/dci21-0034 PMC8929179

[19] Ko JH, Kim TN. Type 2 diabetes remission with significant weight loss: definition and Evidence-Based interventions. J Obes Metab Syndr. 2022;31(2):123–33. 35618657 10.7570/jomes22001 PMC9284579

[20] Kelly J, Karlsen M, Steinke G. Type 2 diabetes remission and lifestyle medicine: A position statement from the American college of lifestyle medicine. Am J Lifestyle Med. 2020;14(4):406–19. 33281521 10.1177/1559827620930962 PMC7692017

[21] Geiker NRW, Astrup A, Hjorth MF, Sjödin A, Pijls L, Markus CR. Does stress influence sleep patterns, food intake, weight gain, abdominal obesity and weight loss interventions and vice versa? Obes Rev. 2018;19(1):81–97. 28849612 10.1111/obr.12603

[22] American Diabetes Association Professional Practice Committee. 3. Prevention or Delay of Type 2 Diabetes and Associated Comorbidities: *Standards of Medical Care in Diabetes—* 2022. Diabetes Care. 2022 45(Supplement_1):S39–45. 10.2337/dc22-S003 34964876

[23] Pot GK, Battjes-Fries MC, Patijn ON, Pijl H, Witkamp RF, De Visser M, et al. Nutrition and lifestyle intervention in type 2 diabetes: pilot study in the Netherlands showing improved glucose control and reduction in glucose Lowering medication. BMJ Nutr Prev Health. 2019;2(1):43–50. 33235957 10.1136/bmjnph-2018-000012 PMC7678479

[24] Pot GK, Battjes-Fries MC, Patijn ON, Van Der Zijl N, Pijl H, Voshol P. Lifestyle medicine for type 2 diabetes: practice-based evidence for long-term efficacy of a multicomponent lifestyle intervention (Reverse Diabetes2 Now). BMJ Nutr Prev Health. 2020;3(2):188–95. 33521528 10.1136/bmjnph-2020-000081 PMC7841830

[25] Action plan for the prevention and control of noncommunicable diseases in the WHO European Region. 2016–2025. [Internet]. 2016 [cited 2025 Feb 28]. Available from: https://www.who.int/europe/publications/i/item/WHO-EURO-2016-2582-42338-58618

[26] De Vries H. An integrated approach for Understanding health behavior; the I-change model as an example. Psychol Behav Sci Int J. 2017;2(2):555585.

[27] Scirocco– Scaling Integrated Care in Context [Internet]. 2018. [cited 2025 Feb 19]. Available from: https://scirocco-project.eu/

[28] Standards for QUality Improvement Reporting Excellence. Promoting Excellence in Health Improvement Reporting. [Internet]. 2020 [cited 2025 Feb 28]. Available from: https://www.squire-statement.org/

[29] Damschroder LJ, Reardon CM, Widerquist MAO, Lowery J. The updated consolidated framework for implementation research based on user feedback. Implement Sci. 2022;17(1):75. 36309746 10.1186/s13012-022-01245-0 PMC9617234

[30] Teoli D, Sanvictores T, An J. SWOT Analysis. In: StatPearls [Internet]. Treasure Island (FL): StatPearls Publishing; 2023 [cited 2025 Feb 19]. Available from: http://www.ncbi.nlm.nih.gov/books/NBK537302/30725987

[31] Powers MA, Bardsley JK, Cypress M, Funnell MM, Harms D, Hess-Fischl A, et al. Diabetes Self-management education and support in adults with type 2 diabetes: A consensus report of the American diabetes association, the association of diabetes care & education specialists, the academy of nutrition and dietetics, the American academy of family physicians, the American academy of PAs, the American association of nurse practitioners, and the American pharmacists association. Diabetes Care. 2020;43(7):1636–49. 32513817 10.2337/dci20-0023

[32] Plan D. Study, Act (PDSA) cycles and the model for improvement. [Internet]. Advancing Quality Alliance (Aqua); [cited 2025 Feb 28]. Available from: https://www.chrome-extension://efaidnbmnnnibpcajpcglclefindmkajhttps://www.dchft.nhs.uk/wp-content/uploads/2022/07/QSIR-Plan-Do-Study-Act.pdf

[33] Ogrinc G, Davies L, Goodman D, Batalden P, Davidoff F, Stevens D. SQUIRE 2.0 (Standards for quality improvement reporting Excellence): Revised Publication Guidelines From a Detailed Consensus Process. J Nurs Care Qual. 2016;31(1):1–8. 26429125 10.1097/NCQ.0000000000000153 PMC5411027

[34] An. inclusive EU4Health programme to better meet the needs of people in Europe. [Internet]. 2025 [cited 2025 Feb 19]. Available from: https://eu4health.eu/

[35] Kalra S, Bantwal G, Kapoor N, Sahay R, Bhattacharya S, Anne B, et al. Quantifying remission probability in type 2 diabetes mellitus. Clin Pract. 2021;11(4):850–9. 34842637 10.3390/clinpract11040100 PMC8628725

[36] Li R, Zhang P, Barker LE, Chowdhury FM, Zhang X. Cost-Effectiveness of interventions to prevent and control diabetes mellitus: A systematic review. Diabetes Care. 2010;33(8):1872–94. 20668156 10.2337/dc10-0843 PMC2909081

